# The effects of berberine on inflammatory markers in Chinese patients with metabolic syndrome and related disorders: a meta‑analysis of randomized controlled trials

**DOI:** 10.1007/s10787-022-00976-2

**Published:** 2022-03-29

**Authors:** Yuqiong Lu, Xiwen Zhang, Jiafang He, Zhanjing Dai, Penghua Shi, Yun Lu, Feng Chang

**Affiliations:** Center for Health Care Policy Research, School of International Pharmaceutical Business, China Pharmaceutical University, 639 Longmian Avenue, Jiangning District, Nanjing, 211198 Jiangsu China

**Keywords:** Berberine, Metabolic syndrome, Inflammatory markers, Meta-analysis

## Abstract

**Background:**

A meta-analysis of randomized controlled trials (RCTs) was conducted to systematically evaluate the effects of berberine on the inflammatory markers of metabolic syndrome (MetS) and related disorders.

**Method:**

Databases that were searched from inception to October 2020 included PubMed, Web of Science, the Cochrane Library, CNKI, VIP, WanFang Data, and ClinicalTrials.gov. Two reviewers independently selected articles and extracted data. The pooled evaluations were entered and analyzed in Review Manager 5.3.

**Results:**

Of the 7387 publications screened, 52 studies were included, and the related trials involved 4616 patients. Pooled estimates showed that the use of berberine could significantly reduce the concentration level of C-reactive protein (CRP) [standardized mean difference (SMD) = − 1.54, 95% confidence intervals (CI) − 1.86, − 1.22, *p* < 0.05], tumor necrosis factor-α (TNF-α) [SMD = − 1.02, 95% CI − 1.27, − 0.77, *p* < 0.05], and interleukin 6 (IL-6) [SMD = − 1.17, 95% CI − 1.53, − 0.81, *p* < 0.05] among patients with MetS and related disorders. However, it did not affect the level of interleukin 1β (IL-1β) [SMD = − 0.81, 95% CI − 1.80, 0.17, *p* = 0.11].

**Conclusion:**

Overall, the use of berberine in patients with MetS and related disorders appeared to significantly decrease several inflammatory markers. Further multi-center and rigorous investigations with larger patient populations are encouraged to confirm the effect of berberine on MetS and related disorders.

## Introduction

Metabolic syndrome (MetS) is a cluster of interconnected physiological and metabolic abnormalities characterized by obesity, insulin resistance, hypertension, and hyperlipidemia (Lee and Herceg 2017). The prevalence of MetS in adults worldwide is reportedly about 20–25% (Ranasinghe et al. 2017). MetS patients have increased risks of cardiovascular disease, diabetes, and some other chronic diseases (Grundy et al. 2006; Arnlöv et al. 2010; Noda et al. 2009). Previous reports have suggested that the development of MetS is associated with increased levels of inflammatory markers, including C-reactive protein (CRP), tumor necrosis factor-α (TNF-α), interleukin 6 (IL-6), interleukin 1 (IL-1), etc. (Festa et al. 2000; Wisse 2004; Akbari et al. 2018; Tabrizi et al. 2018, 2019). Pharmacological strategies to reduce inflammation have become more widespread and more useful in treating MetS and related disorders (Esser et al. 2015).

Berberine is an isoquinoline quaternary alkaloid that can be found in plant extracts produced from Berberis vulgaris and some traditional Chinese medicinal herbs, and it has been found to perform well in managing blood sugar, blood lipids, blood pressure, and without causing serious adverse events (Lan et al. 2015; Liang et al. 2019; Ju et al. 2018). Given that berberine costs less than many other drugs, it could have great potential for use in the management and control of MetS and related disorders. As for the effects of berberine on the concentration level of inflammatory markers, the results of randomized controlled trials (RCTs) have been inconsistent. A systematic review conducted by Beba et al. suggested that berberine could reduce the concentration level of CRP, but only five studies were included in the analysis, and the experimental and control groups of included studies were based on different populations (Beba et al. 2019; Chen et al. 2016; Hu et al. 2012). A more thorough evaluation of the effects of berberine on inflammatory markers in patients with MetS and related disorders needs to be further analyzed with multiple outcomes and evidence from more RCTs. To our knowledge, there are no RCTs relative to this study field in other nations, but many in China. Besides, these RCTs have not been included in systematic reviews or meta-analyses for qualitative or quantitative research.

The present study summarizes a meta-analysis that systematically reviewed and quantified the effects of berberine use on inflammatory markers in Chinese patients with MetS and related disorders to provide special evidence for supporting pharmacists’ and physicians’ clinical actions and decisions in China’s MetS and related disorders management.

## Materials and methods

### Search strategy and study selection

The meta-analysis was conducted based on the recommendations of the Cochrane Collaboration (Higgins et al. 2020), and has been reported according to the Preferred Reporting Items for Systematic Review and Meta-Analysis (PRISMA) guidelines (Page et al. 2021). The databases of PubMed, Web of Science, the Cochrane Library, China National Knowledge Infrastructure (CNKI), VIP Chinese periodical service platform, WanFang Data, and ClinicalTrials.gov (http://www.clinicaltrial.gov) were searched from the date of their inception to October 2020. Medical Subject Headings and text search words included patients [“metabolic syndrome” or “acute coronary syndromes” or “coronary artery disease” or “CVD” or “diabetic” or “T1DM” or “T2DM” or “overweight” or “obese” or “chronic kidney disease” or “end-stage renal disease” or “dialysis” or “heart failure” or “myocardial infarction” or “atherosclerotic” or “hypercholesterolemic” or “hypertension” or “high blood pressure” or “dyslipidemia” or “hyperlipidemia” or “polycystic ovary syndrome” or “stable angina” or “unstable angina” or “diabetic nephropathy” or “obesity” or “stable atherosclerotic plaques” or “atherosclerotic”] (Akbari et al. 2018, 2019; Tabrizi et al. 2018, 2019; Hamedifard et al. 2019), intervention [“berberine”], and outcomes [“CRP” or “IL-6” or “TNF-α” or “IL-1” or “inflammatory”]. References cited by the included studies were traced to uncover relevant additional studies.

### Inclusion and exclusion criteria

All clinical trials that met the following criteria which were defined according to the PICO strategy recommended by Cochrane were included: (1) the study population consisted of Chinese patients diagnosed with MetS and related disorders. The MetS-related disorders included acute coronary syndrome, coronary artery disease, cerebrovascular disease, diabetes, obesity, chronic kidney disease, heart failure, myocardial infarction, atherosclerosis, hypercholesterolemia, hypertension, dyslipidemia, hyperlipidemia, polycystic ovary syndrome, angina pectoris, diabetic nephropathy, and stable atherosclerotic plaques; (2) the experimental group was treated with berberine or berberine combined with other treatments, and placebo or non-berberine treatments were used as the control group; (3) RCTs comparing outcomes in CRP, TNF-α, IL-6, and IL-1.

Studies with the following criteria were excluded from this meta-analysis: (1) duplicate and non-full-text publications; (2) reviews, non-human studies, and retrospective and observational studies; and (3) published in languages other than Chinese or English.

### Data extraction and risk-of-bias assessment

Studies were independently selected by two authors (XWZ and JFH), and they achieved good agreement (*κ* = 0.879). Conflicts between the two authors were resolved by the opinion of a third author (YQL). Eligibility screening was performed in two steps: (1) title and abstract screening for relevance to the study objective, and (2) full-text screening for eligibility for meta-analysis. For each eligible study, the following information was extracted (1) basic information (e.g., first author, year of publication, sample size); (2) baseline characteristics of intervention and study population; and (3) relevant outcomes, including CRP, TNF-α, IL-6, and IL-1. Two authors (XWZ and JFH) independently extracted data from each selected RCTs using a standard abstraction excel sheet (*κ* = 0.962).

The methodological quality of each RCT was evaluated by two independent investigators (XWZ and JFH) using the Cochrane Risk of Bias assessment tool (*κ* = 0.973). The assessment domains of the Cochrane Risk of Bias assessment tool include selection bias, performance bias, detection bias, attrition bias, reporting bias, and other biases (Higgins et al. 2020).

### Statistical analysis

The meta-analysis was undertaken in Review Manager version 5.3 (Cochrane Collaboration, Oxford, UK). Standardized mean differences (SMDs) and 95% confidence intervals (CIs) were used to assess continuous outcomes. *p* values ≤ 0.05 were considered to be statistically significant. Heterogeneity among the included studies was assessed using the *I*^2^ estimate and the *p* value of the Chi-square test. *I*^2^ values < 50% and *p* value > 0.10 were determined to indicate no significant heterogeneity, and the fixed-effect (FE) model was used for meta-analysis. When significant heterogeneity was determined, its source was further evaluated by sensitivity analyses or subgroup analyses. Sensitivity analyses were conducted to assess the effect of each trial on the validity of the pooled overall SMDs using the leave-one-out method. Subgroup analyses were conducted according to the following variables: dosage of berberine (< 0.9 g daily vs. ≥ 0.9 g daily), type of condition (metabolic syndrome vs. type 2 diabetes vs. diabetic nephropathy vs. cardiovascular disease vs. polycystic ovary syndrome vs. other), duration of study (< 3 months vs. ≥ 3 months vs. unclear), and sample size (< 30 vs. 30–60 vs. > 60). In the absence of clinical and methodological heterogeneity, the random-effects (RE) model was used to analyze the outcomes. The results of the meta-analysis were shown in forest plots. Publication bias was detected by funnel plot symmetry tests and Egger’s regression tests. Egger’s regression test was undertaken in Stata /MP version 16.0 (Stata Corp., College Station, TX, USA).

## Results

### Search results

**Fig. 1:**
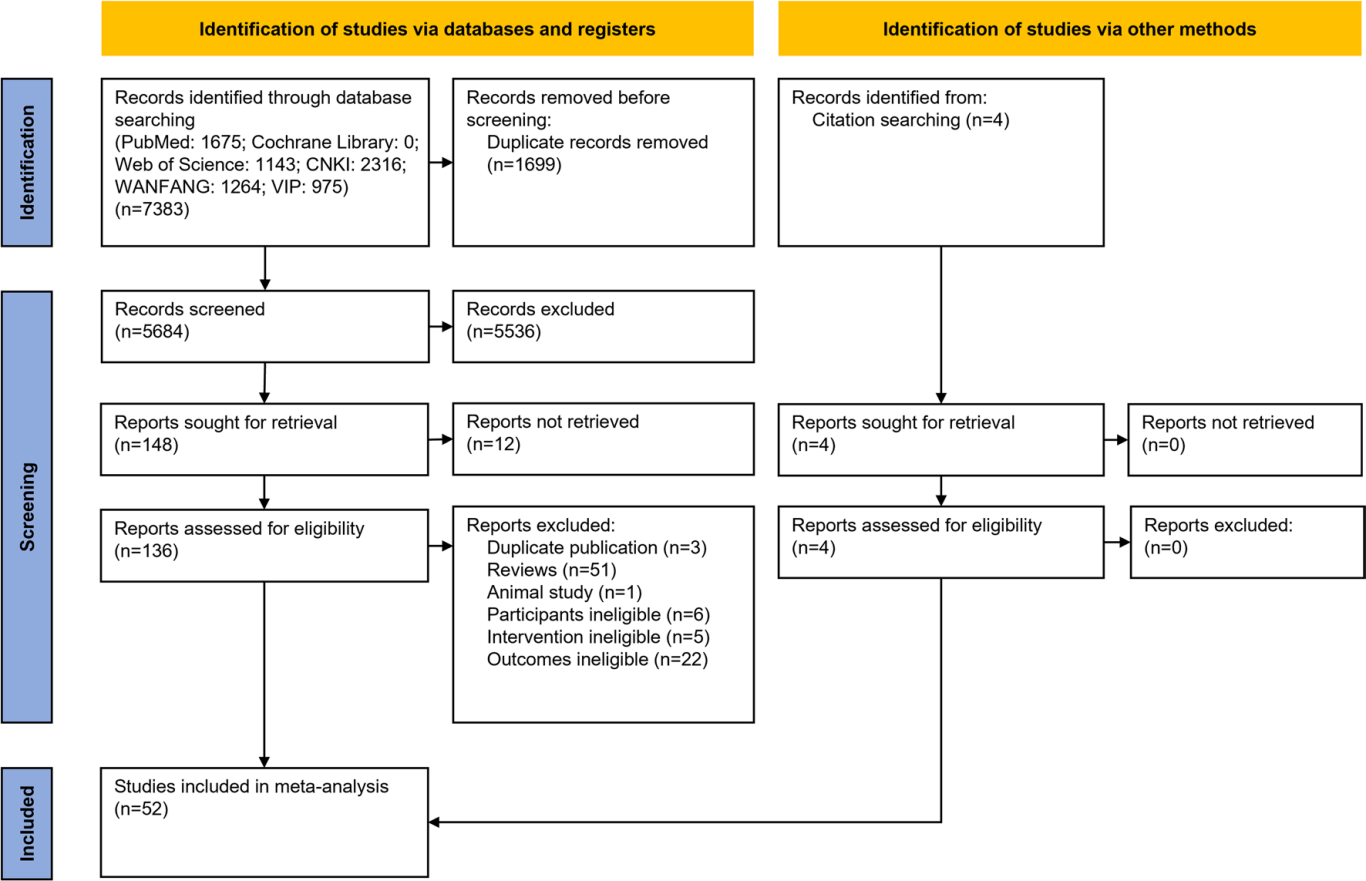
Flowchart of the search, inclusion, and exclusion study selection

A total of 7387 articles were retrieved from the initial search. After screening titles and abstracts, 152 studies were potentially eligible, and these were retrieved for full-text review. After reading the full text, 100 were excluded, because they failed to meet the inclusion criteria. Ultimately, 52 studies that fully satisfied the pre-established inclusion criteria of this meta-analysis were included. The search procedure and reasons for exclusion can be found in the flowchart presented in Fig. 1.

### Study characteristics

The 52 included studies were published between 2008 and 2020 (Liu and Hu 2008; Xu et al. 2008; Zhang et al. 2008, 2010, 2014; Liu et al. 2010; Sheng and Xie 2010; Zhu 2010; Meng et al. 2011; Xiang et al. 2011; Zhou and Huang 2011, 2012; Deng et al. 2012; Dou et al. 2012; Liu and Wang 2012; Yu et al. 2012; Shu 2014; Dai et al. 2015; Chen et al. 2015, 2017; Zhan et al. 2015; Zhu et al. 2015; Li et al. 2016; Sun 2016, 2017; Wang 2016; Zhou et al. 2016; Dong et al. 2017; Li 2017a, b, c Yuan et al. 2017; Bai et al. 2018; Du and Zhang 2018; Fan et al. 2018; He et al. 2018; He 2018; Huang et al. 2018; Li and Deng 2018; Lie et al. 2018a, b Lu et al. 2018; Ning 2018; Wang et al. 2018; Yang et al. 2018, 2020; Cao and Su 2019; Lai et al. 2019; Lan et al. 2019; Xie and Huang 2019; Yang and Yin 2019; Ye and You 2019). The collective patient population comprised 2304 individuals in the experimental group and 2312 individuals in the control group. There were 41 studies that reported the level of CRP, 26 that reported the level of TNF-α, 25 that reported the level of IL-6, and three studies that reported the level of IL-1β. The main characteristics of these studies are presented in Table 1

**Table 1:** Characteristics of included studies

Study		Population		Sample size (*C*/*E*)		Age (years)			Intervention			Duration	Presented data
						*C*	*E*		*C*	*E*			
Liu and Hu (2008)		Type 2 diabetes		30/30		53.07 ± 8.51	52.00 ± 9.81		Metformin 1.5 g/d	Metformin + berberine 0.9–1.5 g/d		8 weeks	CRP
Xu et al. (2008)		Diabetic nephropathy		40/40		51 ± 3.5^a^	51 ± 3.5^a^		Pioglitazone 30 mg/d	Pioglitazone + berberine 0.9 g/d		12 weeks	CRP
Zhang et al. (2008)		Type 2 diabetes and dyslipidemia		58/52		N/A	51 ± 10		Placebo	Berberine 1.0 g/d		3 months	CRP, IL-6
Liu et al. (2010)		Type 2 diabetes		20/20		59.40 ± 15.40	62.80 ± 12.20		Basic treatments^b^	Basic treatments^b^ + berberine 0.9 g/d		3 months	CRP, TNF-α, IL-6
				20/20		64.45 ± 14.40			Basic treatments^b^ + Rosiglitazone 4 mg/d				
Sheng and Xie (2010)		Type 2 diabetes		30/30		51 ± 8	52 ± 11		Glipizide 10 mg/d + metformin 1.5 g/d	Glipizide + metformin + berberine 1.5 g/d		3 months	CRP, TNF-α, IL-1β, IL-6
Zhang et al. (2010)		Acute coronary syndromes		20/20		61.42 ± 8.60^a^	61.42 ± 8.60^a^		Basic treatments^b^	Basic treatments^b^ + berberine 0.9 g/d		30 days	CRP
Zhu (2010)		Diabetic nephropathy		48/44		66.69 ± 8.32	65.71 ± 8.41		Irbesartan 0.15 g/d	Irbesartan + berberine 1.2 g/d		12 weeks	CRP, TNF-α
Meng et al. (2011)		Type 2 diabetes		30/30		53 ± 13.9	51 ± 13.3		Insulin	Insulin + berberine 0.9 g/d		12 weeks	TNF-α, IL-6
Xiang et al. (2011)	Type 2 diabetes		20/20		N/A		N/A	Placebo		Berberine 1.2 g/d		12 weeks	CRP, TNF-α, IL-6
			20/20		N/A			Aspirin 0.1 g/d					
Zhou and Huang (2011)	Hyperlipidemia		60/60		N/A		N/A	No treatment		Berberine 0.9 g/d		4 months	CRP
Deng et al. (2012)	Polycystic ovary syndrome and insulin resistance		28/31		26.75 ± 2.62		25.74 ± 2.66	Ethinylestradiol cyproterone 2 mg: 0.035 mg/d + placebo		Ethinylestradiol cyproterone + berberine 0.9 g/d		3 menstrual cycles	CRP, TNF-α
Dou et al. (2012)	Obesity		60/58		47.68 ± 8.40		48.42 ± 8.60	Vitamin C 0.9 g/d		Berberine 0.9 g/d		4 weeks	CRP
Liu and Wang (2012)	Ischemic heart disease and heart failure		44/50		69.6 ± 8.2		67.5 ± 10.3	Basic treatments^b^		Basic treatments^b^ + berberine 0.9 g/d		8 weeks	TNF-α
Yu et al. (2012)	Type 2 diabetes		24/24		45.6 ± 5.4^a^		45.6 ± 5.4^a^	Glibenclamide 5 mg/d		Exenatide 5 μg/d + berberine 0.9 g/d		12 weeks	CRP
Zhou and Huang (2012)	Obesity and type 2 diabetes		46/46		46.67 ± 8.52^a^		46.67 ± 8.52^a^	Metformin 1.5 g/d		Metformin + berberine 0.6 g/d		12 weeks	CRP
Shu (2014)	Type 2 diabetes		32/32		61.21 ± 13.52		62.80 ± 12.20	Insulin		Insulin + berberine 0.9 g/d		24 weeks	CRP
Zhang et al. (2014)	Cerebral infarction		30/30		54.1 ± 4.6		55.6 ± 5.2	Basic treatments^b^		Basic treatments^b^ + atorvastatin + berberine 0.4 g/d		4 weeks	CRP
			30/30		54.3 ± 4.9			Basic treatments^b^ + atorvastatin 40 mg/d					
Dai et al. (2015)	Hypertension and type 2 diabetes		33/39		53.06 ± 10.36		55.31 ± 11.79	Basic treatments^b^		Basic treatments^b^ + berberine 0.3 g/d		2 years	CRP
Chen et al. (2015)	Coronary artery disease and hypercholesteremia		40/40		51.5 ± 10.4		52.1 ± 9.8	Simvastatin 20 mg/d		Simvastatin 10 mg/d + berberine 0.5 g/d		1 month	CRP
Zhan et al. (2015)	Type 2 diabetes with hyperlipidemia		40/40		51.6 ± 3.8^a^		51.6 ± 3.8^a^	Basic treatments^b^ + metformin 1.5 g/d		Basic treatments^b^ + metformin + berberine 0.6 g/d		3 months	CRP
Zhu et al. (2015)	Acute ischemic stroke		28/16		66.25 ± 8.83		63.31 ± 8.10	Atorvastatin 20 mg/d + aspirin 0.1 g/d		Atorvastatin 20 mg/d + aspirin + berberine 0.4 g/d		3 months	CRP
			11/16		66.45 ± 8.86			Atorvastatin 40 mg/d + aspirin 0.1 g/d					
Li et al. (2016)	Insulin resistance with schizophrenia		33/31		40.18 ± 12.21		40.14 ± 9.40	Risperidone 3.85 ± 0.94 mg/d + placebo		Risperidone 3.77 ± 0.85 mg/d + berberine 0.9 g/d		12 weeks	TNF-α, IL-1β, IL-6
Sun (2016)	Obesity and type 2 diabetes		48/48		52.37 ± 4.48		52.32 ± 4.45	Sitagliptin 0.1 g/d		Sitagliptin + berberine 0.9 g/d		12 weeks	CRP, IL-6
Wang (2016)	Type 2 diabetes		25/25		N/A		N/A	Basic treatments^b^		Basic treatments^b^ + berberine 0.3 g/d		3 months	CRP, IL-6
Zhou et al. (2016)	Obesity and type 2 diabetes		30/30		55.6 ± 12.7		56.4 ± 10.9	Basic treatments^b^		Basic treatments^b^ + berberine 0.6 g/d		3 months	CRP, TNF-α, IL-6
Chen et al. (2017)	Metabolic syndrome with renal damage		10/10		40.20 ± 5.89		38.70 ± 10.3	Losartan 0.1 g/d		Losartan + berberine 0.9 g/d		8 weeks	TNF-α
Dong et al. (2017)	Type 2 diabetes		49/49		51.34 ± 4.43		52.23 ± 4.41	Metformin 1.5 g/d		Metformin + berberine 0.9 g/d		12 weeks	CRP, TNF-α, IL-6
Li (2017a)	Metabolic syndrome with schizophrenia		42/40		42.14 ± 11.61		41.86 ± 10.22	Olanzapine + metformin 0.75 g/d		Olanzapine + berberine 0.9 g/d		12 weeks	TNF-α, IL-1β, IL-6
Li (2017b)	Obesity and type 2 diabetes		30/30		51.24 ± 3.91		50.54 ± 3.78	Sitagliptin 0.1 g/d		Sitagliptin + berberine 0.9 g/d		3 months	CRP, IL-6
Li (2017c)	Acute cerebral ischemic stroke		60/60		61.94 ± 3.77		62.84 ± 4.67	Basic treatments^b^		Basic treatments^b^ + berberine 0.9 g/d		14 days	CRP, IL-6
Sun (2017)	Type 2 diabetes		91/91		58.34 ± 11.21		58.95 ± 10.57	Metformin 1.5 g/d		Metformin + berberine 0.09 g/d		8 weeks	CRP, TNF-α, IL-6
Yuan et al. (2017)	Type 2 diabetes		41/41		65.78 ± 8.96		66.13 ± 9.06	Glimepiride 1 mg/d		Glimepiride + Gegen Qinlian Decoction + berberine 0.6 g/d		2 weeks	CRP, TNF-α
Bai et al. (2018)	Hyperlipidemia		75/75		63.38 ± 7.24		63.29 ± 7.85	Ezetimibe 10 mg/d		Ezetimibe + berberine 0.4 g/d		1 month	CRP
Du and Zhang (2018)	Coronary heart disease		12/18		66 ± 10		60 ± 6	Basic treatments^b^		Basic treatments^b^ + berberine 0.9 g/d		3 months	CRP, TNF-α, IL-6
Fan et al. (2018)	Type 2 diabetes		40/40		52.71 ± 7.89		53.27 ± 8.15	Metformin 1.5 g/d		Metformin + berberine 1.5 g/d		3 months	CRP, TNF-α, IL-6
He et al. (2018)	Diarrhea with hyperlipidemia		62/62		55.16 ± 6.79		56.78 ± 6.74	Basic treatments^b^ + levofloxacin 0.5 g/d		Basic treatments^b^ + berberine 0.36 g/d		8 weeks	CRP, TNF-α, IL-6
He (2018)	Diabetic nephropathy		52/52		56.4 ± 7.3		56.2 ± 7.5	Basic treatments^b^ valsartan 80 mg/d		Basic treatments^b^ + valsartan + berberine 1.2 g/d		12 weeks	CRP, TNF-α
Huang et al. (2018)	Type 2 diabetes		65/65		67.16 ± 8.54		66.09 ± 8.67	Insulin		Insulin + berberine 1.8 g/d		1 month	TNF-α
Li and Deng (2018)	Nonalcoholic fatty liver disease		53/53		74.68 ± 4.32		74.07 ± 5.16	Polyene phosphatidyl choline 1.368 g/d		Polyene phosphatidyl choline + berberine 0.36 g/d		12 weeks	TNF-α
Lie et al. (2018a)	Polycystic ovary syndrome		38/38		N/A		N/A	Ethinylestradiol cyproterone 2 mg: 0.035 mg/d + placebo		Ethinylestradiol cyproterone + berberine 0.9 g/d		21 days	CRP
Lie et al. (2018b)	Type 2 diabetes		57/57		57 ± 12		53 ± 15	Basic treatments^b^		Basic treatments^b^ + berberine 1.2 g/d		6 months	CRP
Lu et al. (2018)	Acute ischemic cerebral infarction		60/60		60.7 ± 5.2		59.9 ± 6.1	Basic treatments^b^ + rosuvastatin 10 mg/d		Basic treatments^b^ + rosuvastatin + berberine 0.9 g/d		N/A	CRP
Ning (2018)	Acute cerebral infarction		39/39		61.00 ± 1.26		60.00 ± 1.47	Basic treatments^b^ + atorvastatin 40 mg/d		Basic treatments^b^ + atorvastatin + berberine 0.9 g/d		15 days	CRP, IL-6
Wang et al. (2018)	Metabolic syndrome with renal damage		10/10		35.62 ± 1.43		37.30 ± 1.96	Basic treatments^b^		Basic treatments^b^ + berberine 0.9 g/d		8 weeks	IL-6
Yang et al. (2018)	Symptomatic atherosclerotic intracranial artery stenosis		60/60		61.98 ± 4.09		61.98 ± 4.09	Simvastatin 40 mg/d + aspirin 0.1 g/d		Simvastatin + aspirin + berberine 1.2 g/d		6 months	CRP
Cao and Su (2019)	Metabolic syndrome and insulin resistance		40/40		65.6 ± 1.8		65.5 ± 1.8	Basic treatments^b^		Basic treatments^b^ + berberine 1.2 g/d		1 month	CRP, TNF-α, IL-6
Lai et al. (2019)	Polycystic ovary syndrome and insulin resistance		48/48		28.48 ± 6.34		29.53 ± 5.21	Metformin 1 g/d		Peikun pills 18 g/d + berberine 0.9 g/d		3 months	CRP, TNF-α, IL-6
Lan et al. (2019)	Hypertensive atherosclerosis		40/40		63.3 ± 6.2		64.2 ± 5.5	Basic treatments^b^		Basic treatments^b^ + berberine 0.9 g/d		8 weeks	TNF-α, IL-6
			40/40				65.1 ± 5.0			Basic treatments + berberine 1.8 g/d			
Xie and Huang (2019)	Diabetic nephropathy		53/53		61.3 ± 1.2		62.1 ± 1.6	Basic treatments^b^ + tripterygium wilfordii polyglycosides 60 mg/d		Basic treatments^b^ + tripterygium wilfordii polyglycosides + berberine 1.5 g/d		90 days	TNF-α, IL-6
Yang and Yin (2019)	Coronary heart disease		30/40		61.37 ± 8.79		60.63 ± 8.53	Basic treatments^b^ + rosuvastatin 10 mg/d		Basic treatments^b^ + berberine 0.9 g/d		4 weeks	CRP, TNF-α
Ye and You (2019)	Acute ischemic cerebral infarction		33/33		56.65 ± 7.12		57.36 ± 6.79	Rosuvastatin 10 mg/d		Rosuvastatin + berberine 0.9 g/d		12 days	CRP, IL-6
Yang et al. (2020)	Type 2 diabetes		96/96		49.7 ± 7.4		49.9 ± 7.8	Metformin 2 g/d		Metformin + berberine 1.5 g/d		3 months	TNF-α, IL-6

*N/A* The date was not reported, *CRP* C-reactive protein, *TNF-α* tumor necrosis factor-alpha, *IL-6* interleukin-6, *IL-1β* interleukin-1 beta, *C* control group, *E* experimental group, *X g/d* X g daily

^a^Only demographic characteristics of the total sample population were reported

^b^Different patients used different drugs for basic treatments

### Risk-of-bias assessment

**Fig. 2:**
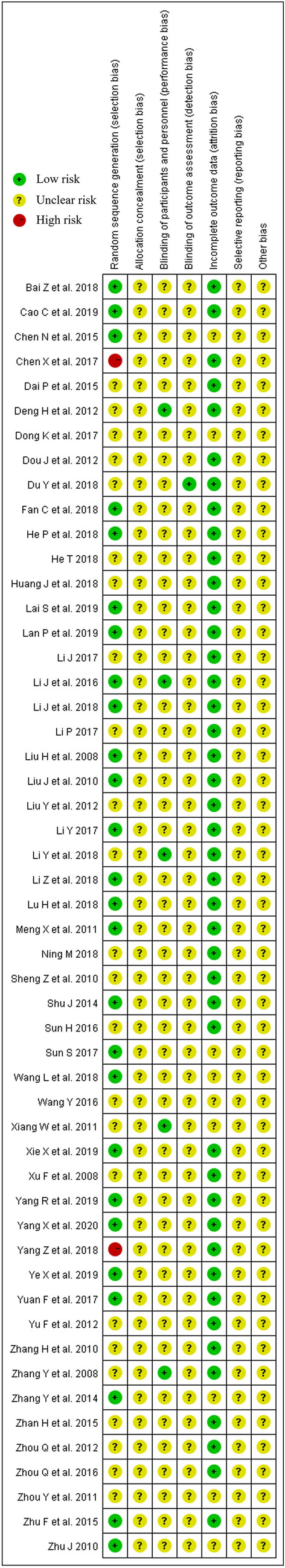
Quality assessment of included studies

Two studies exhibited a high risk of bias in the “random sequence generation” domain (Chen et al. 2017; Yang et al. 2018), since their methods taken to generate random sequences and arrange groups did not accord with the randomization standard. Twenty-four studies exhibited an unclear risk without information about concealment of the allocation sequence. All included studies exhibited an unclear risk in the “allocation concealment” domain because of the lack of detailed description of allocation. Only six studies illustrated the details of blinding (Zhang et al. 2008; Xiang et al. 2011; Deng et al. 2012; Li et al. 2016; Du and Zhang 2018; Li and Deng 2018). Forty-three studies exhibited a low risk of attrition bias without incomplete outcome data. The domain “reporting bias” exhibited an unclear risk of bias, because the measurement of the concentration of inflammatory markers was not mentioned. The domain “other bias” exhibited an unclear risk of bias due to insufficient information. In general, many domains were assessed as “unclear risk”, which indicated that the included studies were likely to be at risk of bias. The risks of bias in each study are summarized in Fig. 2

### Main outcomes

**Fig. 3:**
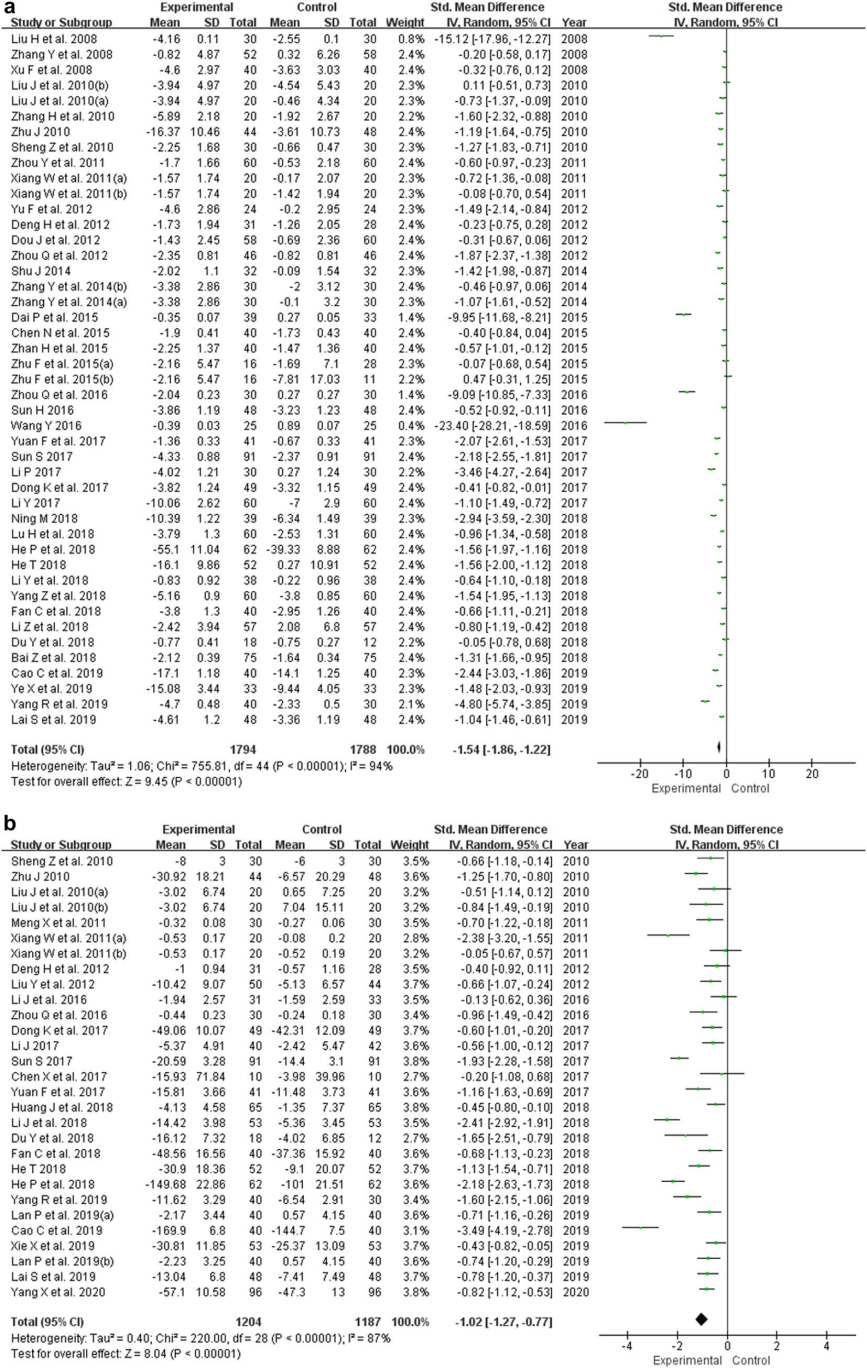

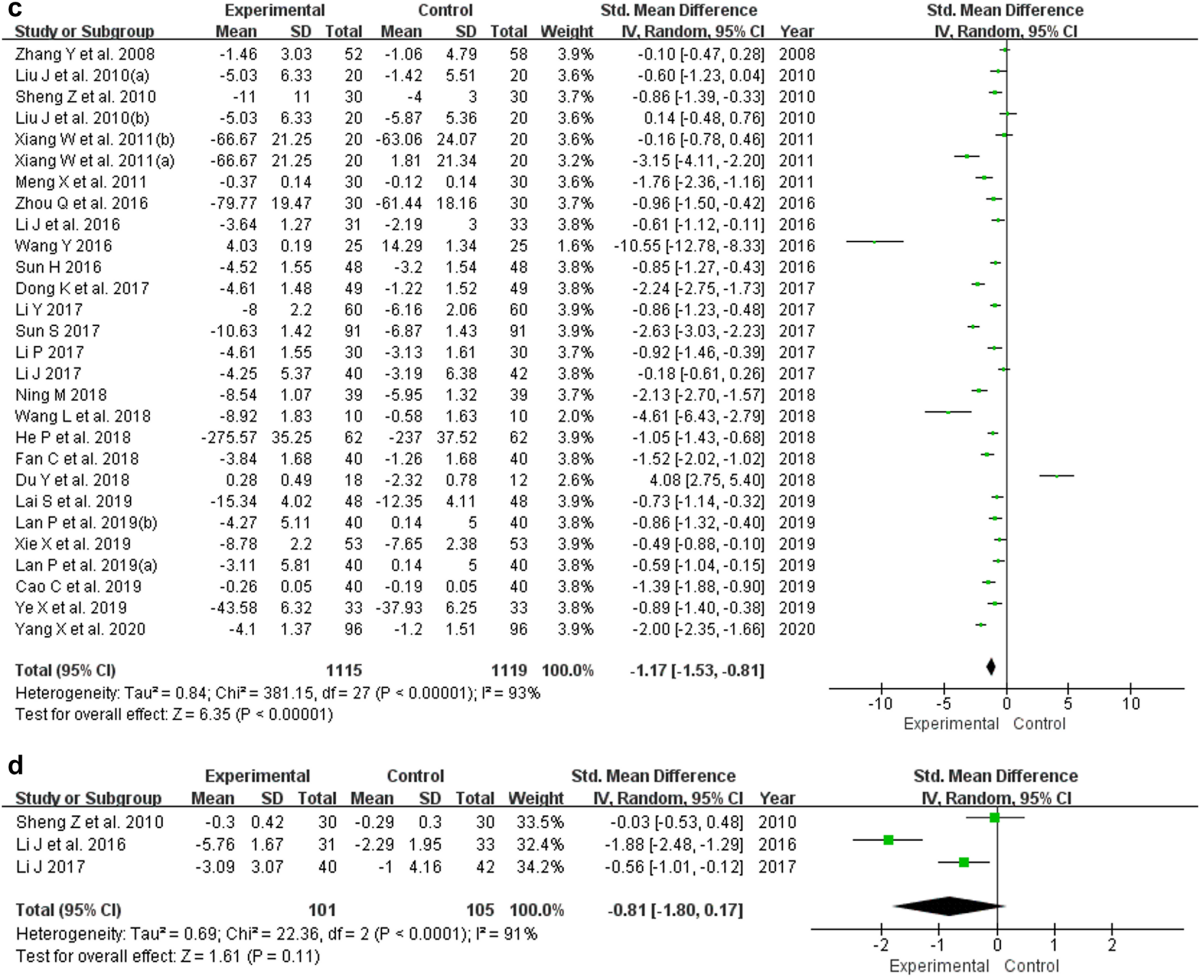
Forest plots of the effect of berberine on **a** CRP, **b** TNF-α, **c** IL-6, and **d** IL-1β. *CRP* C-reactive protein, *TNF-α* tumor necrosis factor-alpha, *IL-6* interleukin-6, *IL-1β* interleukin-1 beta

Forest plots that demonstrate the effects of berberine use on the evaluated inflammatory markers are shown in Fig. 3. The pooled findings using random-effects model showed that berberine use in patients with MetS and related disorders significantly decreased the concentration level of CRP (SMD = − 1.54; 95% CI − 1.86, − 1.22; *p* < 0.05), TNF-α (SMD = − 1.02; 95% CI − 1.27, − 0.77; *p* < 0.05), and IL-6 (SMD = − 1.17; 95% CI − 1.53, − 0.81; *p* < 0.05). Moreover, pooled findings from the random-effects model showed that there was no significant impact of berberine on the level of IL-1β (SMD = − 0.81; 95% CI − 1.80, 0.17; *p* = 0.11).

### Heterogeneity

The meta-analysis showed statistically significant heterogeneity for the outcomes of CRP (*I*^2^ = 94%; *p* < 0.10), TNF-α (*I*^2^ = 87%; *p* < 0.10), IL-6 (*I*^2^ = 93%; *p* < 0.10), and IL-1β (*I*^2^ = 91%; *p* < 0.10), as shown in Fig. 3. Following sensitivity analyses, the heterogeneity did not change significantly and only reduced by 1–4%, with the elimination of individual studies. And there was not any statistically significant difference between before and after sensitivity pooled SMDs for CRP, TNF-α, IL-6, and IL-1β concentration levels, as presented in Table 2.

**Table 2:** Sensitivity analyses of berberine's influence on inflammation

Outcomes	Pre-sensitivity analyses			Upper and lower of effect size	Post-sensitivity analyses		
	No. of studies included	Pooled SMD (RE)	95% CI		Pooled SMD (RE)	95% CI	Excluded studies
CRP	45	− 1.54	− 1.86, − 1.22	Upper	− 1.39	− 1.69, − 1.09	Dai et al. (2015)
				Lower	− 1.58	− 1.90, − 1.26	Zhu et al. (2015)
TNF-α	29	− 1.02	− 1.27, − 0.77	Upper	− 0.94	− 1.17, − 0.72	Cao and Su (2019)
				Lower	− 1.05	− 1.30, − 0.80	Xiang et al. (2011), Li et al. (2016)
IL-6	28	− 1.17	− 1.53, − 0.81	Upper	− 1.02	− 1.35, − 0.69	Wang (2016)
				Lower	− 1.29	− 1.63, − 0.95	Du and Zhang (2018)
IL-1β	3	− 0.81	− 1.80, 0.17	Upper	− 0.31	− 0.84, 0.22	Li et al. (2016)
				Lower	− 1.21	− 2.50, 0.08	Sheng and Xie (2010)

*CRP* C-reactive protein, *TNF-α* tumor necrosis factor-alpha, *IL-6* interleukin-6, *IL-1β* interleukin -1 beta, *SMD* standardized mean differences, *RE*, random effect

Following subgroup analyses, heterogeneity was changed among some of the strata of subgroups. The heterogeneity changed significantly in the strata of polycystic ovary syndrome (*I*^2^ = 19%; *p* = 0.27) and ≥ 3 months (*I*^2^ = 10%; *p* = 0.35) for TNF-α. Furthermore, there were significant differences between before and after subgroup analyses in the stratum of metabolic syndrome for TNF-α (SMD = − 1.42; 95% CI − 3.38, 0.55; *p* > 0.05) and the stratum of cardiovascular disease for IL-6 (SMD = − 0.42; 95% CI − 1.24, 0.39; *p* > 0.05). These results of subgroup analyses suggested that type of condition and duration of study may be the source of heterogeneity in the meta-analysis. Table 3 shows the subgroup analysis of the influence of berberine on CRP, TNF-α, and IL-6.

**Table 3:** Subgroup analyses of the influence of berberine on CRP, TNF-α, and IL-6

Variables	*N*	*I*^2^ (%)	SMD (95% CI)	*p* value
**CRP**				
Total	45	94	− 1.54 [− 1.86, − 1.22]	< 0.00001
Dosage of berberine				
< 0.9 g/d	14	96	− 2.45 [− 3.23, − 1.67]	< 0.00001
≥ 0.9 g/d	31	92	− 1.24 [− 1.56, − 0.93]	< 0.00001
Type of condition				
Metabolic syndrome	1	–-	− 2.44 [− 3.03, − 1.86]	< 0.00001
Type 2 diabetes	21	96	− 2.38 [− 3.02, − 1.75]	< 0.00001
Diabetic nephropathy	3	92	− 1.03 [− 1.75, − 0.30]	< 0.00001
Cardiovascular disease	13	92	− 1.20 [− 1.72, − 0.67]	< 0.00001
Polycystic ovary syndrome	3	65	− 0.65 [− 1.11, − 0.20]	0.005
Other	4	89	− 0.94 [− 1.51, − 0.37]	< 0.001
Duration of study				
< 3 months	25	93	− 1.50 [− 1.88, − 1.12]	< 0.00001
≥ 3 months	19	95	− 1.72 [− 2.32, − 1.12]	< 0.00001
Unclear	1	—	− 0.96 [− 1.34, − 0.58]	< 0.00001
Sample size				
< 30	10	93	− 1.02 [− 1.86, − 0.18]	0.02
30–60	32	95	− 1.71 [− 2.09, − 1.34]	< 0.00001
> 60	3	83	− 1.68 [− 2.21, − 1.16]	< 0.00001
**TNF-α**				
Total	29	87	− 1.02 [− 1.27, − 0.77]	< 0.00001
Dosage of berberine				
< 0.9 g/d	5	84	− 1.74 [− 2.25, − 1.22]	< 0.00001
≥ 0.9 g/d	24	80	− 0.85 [− 1.08, − 0.63]	< 0.00001
Type of condition				
Metabolic syndrome	3	96	− 1.42 [− 3.38, 0.55]	0.16
Type 2 diabetes	13	82	− 0.89 [− 1.19, − 0.58]	< 0.00001
Diabetic nephropathy	3	78	− 0.93 [− 1.44, − 0.41]	0.0004
Cardiovascular disease	5	66	− 1.00 [− 1.39, − 0.60]	< 0.00001
Polycystic ovary syndrome	2	19	− 0.62 [− 0.99, − 0.26]	0.0008
Other	3	94	− 1.58 [− 2.97, − 0.18]	0.001
Duration of study				
< 3 months	19	91	− 1.16 [− 1.52, − 0.80]	< 0.00001
≥ 3 months	10	10	− 0.72 [− 0.86, − 0.57]	< 0.00001
Sample size				
< 30	6	81	− 0.92 [− 1.59, − 0.24]	0.008
30–60	19	86	− 0.98 [− 1.26, − 0.70]	< 0.00001
> 60	4	95	− 1.34 [− 2.12, − 0.55]	0.0009
IL-6				
Total	28	93	− 1.17 [− 1.53, − 0.81]	< 0.00001
Dosage of berberine				
< 0.9 g/d	4	97	− 3.16 [− 4.73, − 1.59]	< 0.0001
≥ 0.9 g/d	24	91	− 0.95 [− 1.29, − 0.61]	< 0.00001
Type of condition				
Metabolic syndrome	3	93	− 1.72 [− 3.19, − 0.25]	0.02
Type 2 diabetes	15	94	− 1.57 [− 2.14, − 1.00]	< 0.00001
Diabetic nephropathy	1	–-	− 0.49 [− 0.88, − 0.10]	0.01
Cardiovascular disease	6	93	− 0.42 [− 1.24, 0.39]	0.31
Polycystic ovary syndrome	1	–-	− 0.73 [− 1.14, − 0.32]	0.0005
Other	2	85	− 0.87 [− 1.29, − 0.44]	0.0002
Duration of study				
< 3 months	16	91	− 1.36 [− 1.78, − 0.95]	< 0.00001
≥ 3 months	12	95	− 0.91 [− 1.57, − 0.26]	0.006
Sample size				
< 30	7	97	− 1.92 [− 3.77, − 0.06]	0.04
30–60	18	83	− 0.98 [− 1.24, − 0.71]	< 0.00001
> 60	3	94	− 1.89 [− 2.76, − 1.02]	< 0.0001

*N* number of SMD included, *CRP* C-reactive protein, *TNF-α* tumor necrosis factor alpha, *IL-6* interleukin-6, *SMD* standardized mean differences, *X g/d* X g daily, *–-* not applicable

### Publication bias

**Fig. 4:**
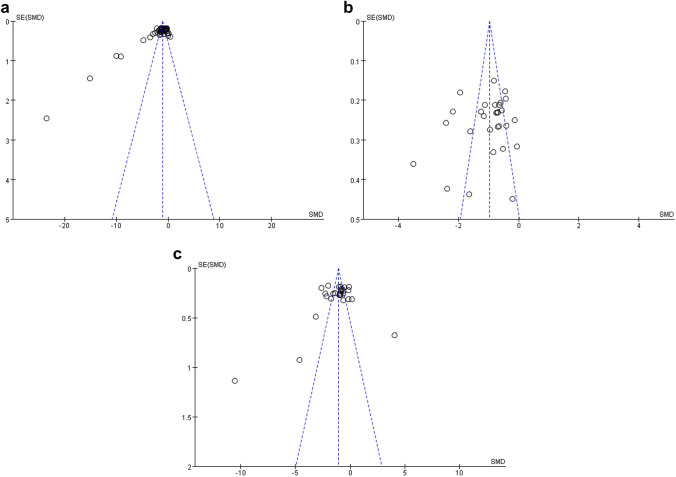
Funnel charts based on **a** CRP, **b** TNF-α, and **c** IL-6. *CRP* C-reactive protein, *TNF-α* tumor necrosis factor alpha, *IL-6* interleukin-6

Funnel plots and Egger’s regression test were not evaluated for IL-1β levels due to the relatively small number of studies with this endpoint. These tests showed no significant evidence of publication bias for meta-analyses assessing the effect of berberine on TNF-α (*p* = 0.46; 95% CI − 1.38, 0.64) and IL-6 (*p* = 0.43; 95% CI − 0.48, 1.09) concentration levels. However, as shown in Fig. 4, the asymmetry displayed in the funnel plot, and Egger’s test (*p* < 0.05; 95% CI 1.27, 2.26) of CRP indicated some publication bias, which probably is attributed to unpublished studies with negative findings.

## Discussion

Regulating inflammatory markers through various pathways to exert anti-inflammatory effects is one possible mechanism of action that berberine may have in the treatment of MetS and related disorders. An animal experiment conducted by Jeong HW found that berberine can restore damaged islet cells by activating the adenosine monophosphate-activated protein kinase (AMPK) signaling pathway (Jeong et al. 2009). In the adipose tissue of obese mice, berberine was shown to significantly down-regulate the expression of pro-inflammatory genes, including IL-1β, IL-6, TNF-α, monocyte chemoattractant protein-1 (MCP-1), inducible nitric oxide synthase (iNOS), and cyclooxygenase 2 (COX-2), and continually inhibit peritoneal macrophages and RAW264.7 cell pro-inflammatory genes (IL-1β, IL-6, iNOS, MCP-1, COX-2, and alkaline metalloproteinase-9) expression induced by lipopolysaccharide (LPS) (Jeong et al. 2009). Additionally, berberine can reduce the phosphorylation of MAPK by intervening in the activation of TNF-α and other inflammatory markers on MAPK (Li et al. 2014). Wan Q reported that berberine inhibits the activation of the extracellular-signal-regulated kinase (ERK) signaling pathway, and down-regulates the expression of TNF-α and IL-6, through in-vitro experiments on human umbilical vein endothelial cells (HUVECs) (Wan et al. 2014). Inflammatory markers like IL-6 and IL-1β regulate and induce the expression of CRP. Furthermore, an increase in CRP levels can facilitate those inflammatory markers when inflammation occurs (Yang et al. 2012).

As an extract from traditional Chinese herbs, berberine has a long history of clinical application and many efficacy trials on humans in China. The meta-analysis included 52 RCTs involving 4616 Chinese patients with MetS and related disorders, which complemented the evidence of the effects of berberine use on inflammatory markers in humans and in China. The results suggested that berberine could reduce the concentration level of CRP significantly, which was consistent with the results of a previous study (Beba et al. 2019). Furthermore, this meta-analysis analyzed three other important inflammatory markers of metabolic syndrome (MetS) and related disorders. The results suggested that berberine could reduce the concentration level of TNF-α, and IL-6 significantly, but could not reduce the concentration level of IL-1β. Sensitivity analyses and subgroup analyses indicated that the results of the meta-analysis were relatively stable. The type of condition had the greatest impact on the heterogeneity and pooled estimates of the meta-analysis. However, due to the small number of included studies and the estimated heterogeneity, there were additional doubts about the pooled estimate result of IL-1β, which need to be resolved in further trials.

There are a few limitations to this meta-analysis. First, the result of the risk-of-bias assessment presented a large proportion of uncertain risks for insufficient information in trial methods. To a certain extent, the potential differences in the methods of random sequence generation, allocation concealment, and concentration measurement among the included studies have caused the high heterogeneity of the meta-analysis results. Second, the study population of all the included studies was Chinese patients, and the sample size of individual clinical trial was small. The results of this meta-analysis are accordingly hard to extrapolate to other ethnic populations or geographical regions. Therefore, more studies with larger sample size, ideally multi-centers, and rigorous design are needed to confirm the effect of berberine on the inflammatory markers of MetS and related disorders.

## Conclusion

Despite the limitations of meta-analysis, the robust methodology followed in selecting RCTs for inclusion and in completing the evaluation does facilitate the conclusion that berberine use in patients with MetS and related disorders appears to have significantly decreased inflammatory markers, including CRP, TNF-α, and IL-6. This study provides new and useful evidence for supporting clinical medication decisions for MetS and related disorders and encourages undertaking further RCTs.

## Data Availability

All data generated or analyzed during this study are included in this published article.
